# Analysis of drug-induced interstitial lung disease caused by herbal medicine using the Japanese Adverse Drug Event Report database

**DOI:** 10.1186/s12906-024-04428-y

**Published:** 2024-03-14

**Authors:** Keita Oura, Mizuki Tanaka, Kiyoka Matsumoto, Riko Satake, Misaki Inoue, Yu Yoshida, Wataru Wakabayashi, Shiori Hasegawa, Mari Iwata, Takaaki Suzuki, Mika Maezawa, Satoshi Nakao, Jun Liao, Kazuhiro Iguchi, Mitsuhiro Nakamura

**Affiliations:** 1 Laboratory of Drug Informatics, Gifu Pharmaceutical University, 1-25-4, Daigaku-Nishi, Gifu, 501-1196 Japan; 2 Kaneichi Pharmaceutical, Company, Limited, Osaka, Japan; 3 Kifune Pharmacy, Gifu, Japan; 4 Gifu Prefectural Government, Gifu, Japan; 5 Department of Pharmacy, Kyushu University Hospital, Fukuoka, Japan; 6 Department of Information Science and Information System, China Pharmaceutical University, Nanjing, China; 7 Laboratory of Community Pharmacy, Gifu Pharmaceutical University, Gifu, Japan

**Keywords:** Drug-induced interstitial lung disease, Herbal medicine, Japanese Adverse Drug Event Report database, JADER, Scutellariae radix

## Abstract

**Background:**

Drug-induced interstitial lung disease (DIILD) is a severe adverse event leading to morbidity and mortality. This study evaluated the adverse event indicators of DIILD and time-to-onset profiles following the daily intake of herbal drugs (*Scutellariae radix* [“ogon” in Japanese], *Bupleuri radix* [“saiko” in Japanese], and *Pinelliae tuber* [“hange” in Japanese]) using the Japanese Adverse Drug Event Report database. DIILD was defined in accordance with the Medical Dictionary for Regulatory Activities.

**Methods:**

The Japanese Adverse Drug Event Report database contained 830,079 reports published between April 2004 and April 2023. The association between herbal medicines and DILLD was evaluated using the pharmacovigilance index as the reporting odds ratio (ROR), logistic regression models, propensity score-matching techniques, and Weibull shape parameters.

**Results:**

The adjusted RORs using multivariate logistic regression models for *Scutellariae radix* (daily intake), *Pinelliae tuber* (daily intake), sex (male), age (≥ 60 years), *Scutellariae radix* (daily intake)*age (≥ 60 years), and *Scutellariae radix* (daily intake)* *Pinelliae tuber* (daily intake) were 1.47 (1.36 − 1.59), 1.05 (1.01 − 1.10), 1.45 (1.34 − 1.57), 1.92 (1.74 − 2.11), 3.35 (3.12 − 3.60), and 1.49 (1.46 − 1.53), respectively. DIILD onset profiles were evaluated using the Weibull shape parameter. A logistic plot of daily intake and onset of DIILD was drawn. ROR signals were detected in 32 of 54 herbal medicines, including *Scutellariae radix*, *Bupleuri radix*, and *Pinelliae tuber*. The median duration (days) (interquartile range) to DIILD onset was 36.0 (27.0–63.0) for Saikokaryukotsuboreito, 35.0 (21.0–55.0) for Saireito, and 31.0 (13.5–67.5) for Shosaikoto. The Weibull shape parameter beta (95% confidence interval) values for Saikokaryukotsuboreito, Saireito, and Shosaikoto were 1.36 (1.08–1.67), 1.36 (1.20–1.52), and 1.31 (0.98–1.68), respectively.

**Conclusions:**

DIILD demonstrated a dose-dependent to crude drugs. Clinicians should strive for the early detection of DIILD and avoid the inadvertent administration of herbal medicines.

## Background

Drug-induced interstitial lung disease (DIILD) is the most common drug-induced lung toxicity that occurs when drug exposure causes inflammation and, eventually, fibrosis of the lung interstitium [1, 2]. DIILD is a severe adverse event (AE) that leads to increased morbidity and mortality rates. In recent years, DIILD has been reported as a risk factor for coronavirus disease 2019 [3]. Therefore, all healthcare professionals should be familiar with potential DIILD as early detection is important to prevent its progression.

Drugs known to cause DIILD include amiodarone, interferons, anticancer drugs (e.g., gefitinib), and Japanese herbal medicines “Kampo” (e.g., Shosaikoto and Saireito) [4]. Kampo is formulated from natural agents, and in Japan, 148 “Kampo extract formulations for prescription” have been approved for ethical use [5, 6]. The percentage of events related to lung injury was 27.8% [7]. Tsukiyama et al. reported a clinical case of DIILD caused by Shosaikoto in 1989 [8], and numerous other clinical cases have been reported. Saikozai is a general term for Japanese herbal medicines mainly containing *Scutellariae radix* (SR, “ogon” in Japanese) and *Bupleuri radix* (BR, “saiko” in Japanese) (e.g., Shosaikoto and Saikokeishikankyoto) and has been reported to cause DIILD [9–13].

Although Kampo is a mixture of multiple crude drugs, other crude drugs, such as SR, BR, and *Pinelliae tuber* (PT, “hange” in Japanese), show positive results in the lymphocyte stimulation test and are thought to be associated with DIILD [8, 11–13]. Both SR and BR are thought to cause lung injury [14]. According to a review by Enomoto et al., the most common formulas are Shosaikoto (26%), Saireito (16%), Seishinrenshiin (8%), and Bofutsushosan (8%) [15]. SR occurred in 86% of the formulas, BR in 59%, and PT in 58% [15]. Furthermore, the Ministry of Health, Labour, and Welfare of the Government of Japan has issued “Pharmaceuticals and Medical Devices Safety Information” on DIILD caused by herbal medicines [16, 17]. However, few studies have examined the relationship between the risk of DIILD and each herbal ingredient or its contents. Therefore, we focused on the constituents of herbal medicines, such as SR, BR, and PT.

Although the risk of DIILD has been described in several studies, this information does not reflect the complexities in real-world clinical practice. Spontaneous reporting systems (SRSs), such as the Japanese Adverse Drug Event Report (JADER) database of the Pharmaceuticals and Medical Devices Agency (PMDA), have been used for pharmacovigilance assessments. The JADER database is a valuable tool for postmarketing surveillance that reflects real-world settings.

In this study, we evaluated the relationship between herbal medicines and DIILD using data from the JADER database. We evaluated AE signals using a pharmacovigilance index known as the reporting odds ratio (ROR), which is currently employed by the Japanese PMDA. We assessed the possible relationships between age, sex, and daily SR, BR, and PT intakes using adjusted RORs and propensity score (PS) matching techniques. Furthermore, we assessed the time-to-onset profiles of DIILD associated with herbal medicines.

## Methods

**Fig. 1:**
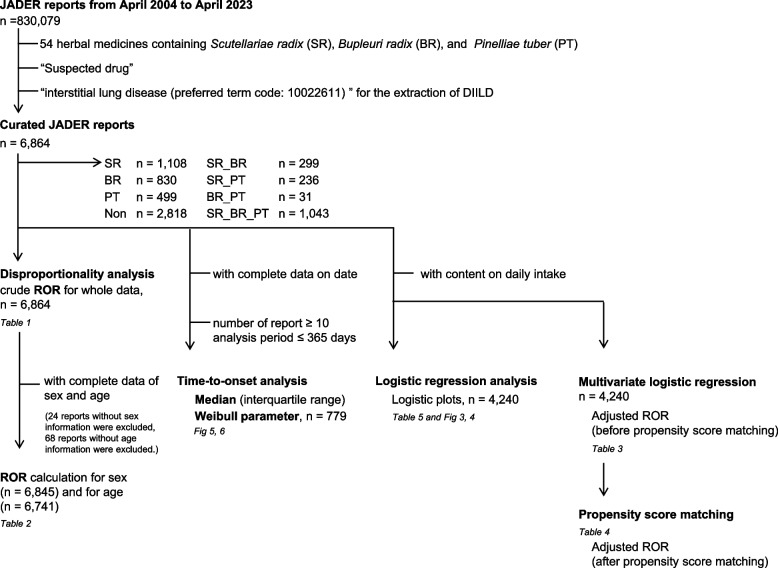
Flowchart of data analysis

Figure 1 summarizes the workflow of downloading JADER data from the PMDA website, excluding reports that were unsuitable for analysis due to missing values, and finally providing the data for analysis (Fig. 1). The JADER database provides information on cases reported by pharmaceutical corporations or medical institutions since 2004, based on the Act on Securing Quality, Efficacy, and Safety of Products Including Pharmaceuticals and Medical Devices (Act No. 145 of 1960), and cases reported by medical institutions since 2013, based on the Immunization Law. Healthcare professionals, marketing approval holders, patients, and consumers voluntarily report adverse events to the PMDA. All data from the JADER database are cleaned and fully anonymized by the regulatory authority of Japan. These data are provided in CSV format, are made publicly available, and can be downloaded from the PMDA website (https://www.pmda.go.jp) [18]. When additional information or corrections are reported in the CSV file, the PMDA updates the appropriate information accordingly. The data in the CSV file are not evaluated by the PMDA with regards to the association between individual drugs and adverse events. The database comprised four tables of data: patient demographic information, drug information, AEs, and primary illness. We integrated a relational database based on the four tables using FileMaker Pro 12 software (FileMaker, Inc., Santa Clara, CA, United States) according to the ASCII Entity Relationship Diagram, which is publicly available on the PMDA website [18]. Drug data included the following role codes assigned to each drug according to its association with AEs: “suspected,” “concomitant,” and “interacting drugs.” We extracted and analyzed the “suspected drug” records.

We listed 147 Kampo extract products approved by the Ministry of Health, Labor, and Welfare [19] and selected 54 herbal medicines containing SR, BR, or PT (Fig. 1). Regardless of whether Kampo included SR, BR, or PT, we classified Kampo into 2^3 = 8 groups. We analyzed the signals for seven groups, excluding those that did not include the three types of crude drugs. When a report contained several Kampo, we counted them as long as the Kampo were in the same group (e.g., a combination of Bofutsushosan [SR] and Orengedokuto [SR] was counted as one case; a combination of Bofutsushosan [SR] and Yokukansan [BR] was not counted as one case; and a combination of Bofutsushosan [SR] and Otsujito [SR_BR] was not counted as one case). When analyzing signals for sex and age, we eliminated incomplete reports (e.g., missing, unknown, and aged) on sex and age from the subsets (Fig. 1).

To extract cases of DIILD, we used “interstitial lung disease” as the preferred term (code:10,022,611) following the terminology preferred by the Medical Dictionary for Regulatory Activities (MedDRA) (version 23.0) [20]. According to the Introductory Guide MedDRA Version 23.0, a preferred term is defined as a clear descriptor (a single medical concept) regarding symptoms, signs, disease, diagnosis, therapeutic indication, and surgical or medical procedure [21]. The contributors only reported AEs according to the International Council for Harmonisation of Technical Requirements for Pharmaceuticals for Human Use (ICH) E2B and international safety reporting guidelines, and relied on the definitions provided by MedDRA.

**Fig. 2:**
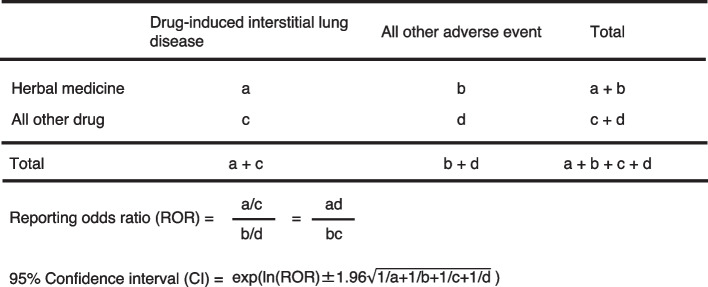
Two-by-two used for the calculation of the reporting odds ratio

ROR is the ratio of the odds of reporting DIILD versus all other AEs for a given herbal medicine to the reporting odds for all other herbal medicines or drugs present in the database [22, 23]. The ROR was calculated using a two-by-two contingency table, where “a” is the number of AE reports in which patients received the herbal medicine and manifested DIILD; “b” is the number of AE reports in which patients received herbal medicine but did not present DIILD; “c” is the number of AE reports in which patients did not receive herbal medicine of interest and had DIILD; and “d” is the number of AE reports in which patients did not receive herbal medicine of interest and did not present DIILD (Fig. 2). In the case of the worked example, it is the ratio of the odds of DIILD divided by the odds of all other AEs (ROR of “Bofutsushosan,” (103/36745)/(307/793334) = 7.3, Table 1). RORs were expressed as point estimates with 95% confidence intervals (CIs). For signal detection, general qualitative judgments are viable; detection of a signal depends on whether the signal indices exceed predefined thresholds, and crude ROR values < 1 indicate no potential exposure-event associations. Crude ROR is an indicator of AEs when the lower limit of the 95% CI is > 1 and the reported number of AEs is > 2 [22, 23].

**Table 1:** Number of reports and ROR of drug-induced interstitial lung disease for each herbal medicine for analysis of whole data

Crude drug component	Herbal medicine	Total (n)	Case (n)	Non-case (n)	ROR (95% CI)
Total		830079	36745	793334	
*Scutellariae radix* (SR)		1051	308	743	9.0 (7.9 − 10.3)
(Ougon)	Bofutsushosan	410	103	307	7.3 (5.8 − 9.1)
	Orengedokuto	185	44	141	6.7 (4.8 − 9.5)
	Shin'iseihaito	110	27	83	7.0 (4.6 − 10.8)
	Seishinrenshiin	99	51	48	23 (15.5 − 34.1)
	Junchoto	46	25	21	25.7 (14.4 − 45.9)
	Unseiin	42	13	29	9.7 (5.0 − 18.6)
	Seihaito	34	11	23	10.3 (5.0 − 21.2)
	Seijobofuto	28	1	27	—
	Ryutanshakanto	24	9	15	13.0 (5.7 − 29.6)
	Sammotsuogonto	23	11	12	19.8 (8.7 − 44.9)
	Nyoshinsan	22	3	19	3.4 (1.0 − 11.5)
	Gorinsan	21	6	15	8.6 (3.3 − 22.3)
	San'oshashinto	6	4	2	43.2 (7.9 − 235.7)
	Ogonto	1	0	1	—
*Bupleuri radix* (BR)		796	86	710	2.6 (2.1 − 3.3)
(Saiko)	Yokukansan	418	42	376	2.4 (1.8 − 3.3)
	Hochuekkito	181	32	149	4.6 (3.2 − 6.8)
	Kamishoyosan	153	7	146	1.0 (0.5 − 2.2)
	Kamikihito	21	3	18	3.6 (1.1 − 12.2)
	Jumihaidokuto	19	2	17	2.5 (0.6 − 11.0)
	Shimpito	2	0	2	—
	Shigyakusan	1	0	1	—
	Jiinshihoto	1	0	1	—
*Pinelliae tuber* (PT)		482	77	405	4.1 (3.2 − 5.2)
(Hange)	Rikkunshito	158	13	145	1.9 (1.1 − 3.4)
	Bakumondoto	131	31	100	6.7 (4.5 − 10.0)
	Shoseiryuto	110	20	90	4.8 (3.0 − 7.8)
	Hangekobokuto	45	10	35	6.2 (3.1 − 12.5)
	Chotosan	14	1	13	—
	Hangebyakujutsutemmato	7	1	6	—
	Unkeito	6	0	6	—
	Bukuryoingohangekobokuto	5	0	5	—
	Goshakusan	3	0	3	—
	Shohangekabukuryoto	1	0	1	—
	Nichinto	1	0	1	—
	Ryokankyomishingeninto	1	1	0	—
	Orento	0	0	0	—
	Jinsoin	0	0	0	—
	Tokito	0	0	0	—
SR_BR		277	115	162	15.4 (12.1 − 19.5)
	Otsujito	154	82	72	24.6 (17.9 − 33.8)
	Saikokeishikankyoto	78	23	55	9.0 (5.5 − 14.7)
	Keigairengyuoto	41	8	33	5.2 (2.4 − 11.3)
	Saikoseikanto	4	2	2	21.6 (3.0 − 153.1)
SR_PT		231	102	129	17.1 (13.2 − 22.2)
	Hangeshashinto	190	81	109	16.1 (12.0 − 21.4)
	Nijutsuto	41	21	20	22.7 (12.3 − 41.8)
BR_PT		31	3	28	2.3 (0.7 − 7.6)
	Yokukansankachimpihange	28	2	26	1.7 (0.4 − 7.0)
	Chikujountanto	3	1	2	—
SR_BR_PT		998	383	615	13.6 (11.9 − 15.4)
	Saireito	456	191	265	15.6 (13.0 − 18.8)
	Saikokaryukotsuboreito	164	50	114	9.5 (6.8 − 13.2)
	Shosaikoto	107	58	49	25.6 (17.5 − 37.4)
	Saibokuto	93	29	64	9.8 (6.3 − 15.2)
	Saikokeishito	85	23	62	8.0 (5.0 − 12.9)
	Daisaikoto	64	25	39	13.8 (8.4 − 22.9)
	Shosaikokakikyosekko	25	7	18	8.4 (3.5 − 20.1)
	Daisaikotokyodaio	4	0	4	—
	Saikanto	0	0	0	—

Number of cases < 2

*ROR* reporting odds ratio, *CI* Confidence interval

We used only reports with complete or calculable data on sex, age, and daily intake of each crude drug. The effectiveness of the explanatory variables was evaluated using a stepwise method with a significance level of 0.05 (forward and backward) [24]. We input the reporting year parameter into the following formula and evaluated the influence of the explanatory variable using the likelihood ratio test:

1 $$\begin{array}{l}\text{log}\left(odds\right)={\mathrm\beta}_{10}+{\mathrm\beta}_{11}Y+{\mathrm\beta}_{12}D1+{\mathrm\beta}_{13}D2+{\mathrm\beta}_{14}S+{\mathrm\beta}_{15}A+{\mathrm\beta}_{16}D1\ast A+{\mathrm\beta}_{17}D1\ast D2\\(\mathrm D1\;=\;\mathrm{daily}\;\mathrm{intake}\;\lbrack\mathrm{SR}\rbrack,\;\mathrm D2\;=\;\mathrm{daily}\;\mathrm{intake}\;\lbrack\mathrm{PT}\rbrack,\;\mathrm S\;=\;\mathrm{sex}\;\lbrack\mathrm{male}\;=\;1,\;\mathrm{female}\;=\;0\rbrack,\;\mathrm A\;=\;\mathrm{age}\;\lbrack\geq60\;\mathrm{years}\;=\;1,\;<60\;\mathrm{years}\;=0\rbrack)\end{array}$$

PS matching was used as an assessment approach to reduce selection bias. PS matching is a statistical matching technique used to construct matched sets with similar covariate distributions without requiring close or exact matches for all individual variables [25–27]. Sex, age, and daily crude drug intake were included in multivariate logistic regression analysis. The presence or absence of DIILD was also assessed. Nearest-neighbor matching was performed based on the calculated PS between the presence and absence of crude drugs. The caliper width of 0.2 of the standard deviation of the PS logit was used. The standard mean difference (SMD) was used as a covariate balance indicator between the presence and absence of crude drugs. An SMD value less than 0.1 was regarded as balanced.

Using a logistic regression model, we estimated the relationship between the daily intake of crude drugs (SR, BR, and PT) and DIILD onset. All of the products used in this analysis are granule “Kampo extract formulations for prescription.” The amount of Kampo extract formulation prescribed per package was specified for each crude drug. For example, two packages (3.75 g) of Shosaikoto granules contained 2.25 g of Shosaikoto extract. Bupleurum Root, Pinellia Tuber, Scutellaria Root, Jujube, Ginseng, Glycyrrhiza, and Ginger were present in the Shosaikoto extract at proportions of 3.5, 2.5, 1.5, 1.5, 1.0, and 0.5, respectively. The daily intake of each crude drug was calculated based on the amount of herbal medicine intake in the JADER reports and information on the package insert of each herbal medicine. The following formula is used:

2 $$\begin{array}{c}\text{log}\left(\mathit{odds}\right)=\beta_{20}+\beta_{21}D\\(\text{D}\hspace{0.17em}=\hspace{0.17em}\mathrm{dailyintake}\lbrack\text{SR},\mathrm{BR},\mathrm{PT}\rbrack)\end{array}$$

The receiver operating characteristic (ROC) curve determined the accuracy of model predictions for treatment allocation. For the ROC curve, the horizontal axis shows the false-positive fraction (1-specificity) and the vertical axis shows the true-positive fraction (sensitivity); the cut-off point has the highest sensitivity while maintaining high specificity.

The period from the prescription of specific drugs to the occurrence of a specific AE was evaluated using median time, quartile, and Weibull shape parameters (WSP) [28]. Duplicate prescriptions for the same patient and reports without a complete date of drug administration or AE onset were excluded. Truncation should be considered when estimating the time to onset of AEs using SRS data. We chose an analysis period of 365 days after the start of drug administration to focus on the onset of AEs within one year after drug administration. The occurrence rate of AEs after prescription depends on the causal mechanism, and often varies over time. In contrast, AEs not associated with the drug occurred at a constant rate.

Median, quartile, and WSP tests were used to evaluate the time-to-onset data. Reports that did not include complete AE occurrences or prescription start times were excluded. Time-to-onset data were calculated using the start date of drug administration and the date that the AE occurred. It is necessary to consider truncation when estimating the time-to-onset of adverse events from JADER data. We selected an analysis period of 365 days after the start of drug administration to focus on the onset of AEs within a year after drug administration. We applied a Weibull distribution to the survival analysis. In this study, we did not use a proportional hazard model or a parametric modeling method such as an accelerated failure time model. The WSP test was used for the statistical analysis of the time-to-onset data and can describe the non-constant ratio of the incidence of AEs. The scale parameter α and shape parameter β determine the scale and shape of the distribution function, respectively. The shape parameter β of the Weibull distribution indicates the hazard without a reference population. If β = 1, then the hazard is estimated to be constant over time (random failure type). If β > 1 and the 95% CI of β excludes the value of 1, the hazard is estimated to increase over time (wear-out failure type). Finally, if β < 1 and the 95% CI β excludes value 1, the hazard is estimated to decrease over time (initial failure type) [29, 30].

Data analysis was performed using the JMP version 11.0 software (SAS Institute, Inc., Cary, NC, USA).

## Results

The JADER database contains 830,079 reports published between April 2004 and April 2023. The ROR signals, including SR, BR, and PT, were detected in 32 of the 54 herbal medicines. The ROR (95% CI) for SR, BR, PT, and the combination of SR, BR, and PT (SR_BR_PT) were 9.0 (7.9 − 10.3), 2.6 (2.1 − 3.3), 4.1 (3.2 − 5.2), and 13.6 (11.9 − 15.4), respectively (Table 1). After excluding missing reports, the number of reports for sex and age were 6,845 and 6,741, respectively. The reported number of male, female, < 20 years, the 20 s, 30 s, 40 s, 50 s, 60 s, 70 s, and ≥ 80 s were 808, 719, 6, 7, 16, 50, 153, 385, 534, and 354, respectively (Table 2).

**Table 2:** Number of reports and ROR of drug-induced interstitial lung disease for sex and age

Category		Total (n)	Case (n)	Non-case (n)	ROR (95% CI)
Sex		6845	1527	5318	
	Male	2560	808	1752	10.2 (9.3 − 11.0)
	Female	4285	719	3566	4.4 (4.1 − 4.8)
Age		6741	1505	5236	
	< 20 years	172	6	166	0.8 (0.3 − 1.8)
	20 s	200	7	193	0.8 (0.4 − 1.7)
	30 s	477	16	461	0.7 (0.5 − 1.2)
	40 s	617	50	567	1.9 (1.4 − 2.5)
	50 s	892	153	739	4.5 (3.8 − 5.3)
	60 s	1276	385	891	9.4 (8.4 − 10.6)
	70 s	1657	534	1123	10.4 (9.4 − 11.5)
	80 s or older	1450	354	1096	7.0 (6.2 − 7.9)

Number of cases < 2

*ROR* Reporting odds ratio, *CI* Confidence interval

The adjusted RORs for SR (daily intake), PT (daily intake), sex (male), age (≥ 60 years), SR (daily intake)*age (≥ 60 years), and SR (daily intake)*PT (daily intake) were 1.47 (1.36 − 1.59), 1.05 (1.01 − 1.10), 1.45 (1.34 − 1.57), 1.92 (1.74 − 2.11), 3.35 (3.12 − 3.60), and 1.49 (1.46 − 1.53), respectively (Table 3).

**Table 3:** The statistics of multivariate logistic regression of daily intake and onset of drug-induced interstitial lung disease

	Estimate (95% CI)		*p*–value (Prob > ChiSq)	Adjusted reporting odds ratio (95% CI)	Crude reporting odds ratio (95% CI)
Intercept	β_10_	-22.62 (-52.73 − 7.46)	0.1406		
Reporting year	β_11_	0.01 (0.00 − 0.03)	0.1735	1.01 (1.00 − 1.03)	
*Scutellariae radix* (SR) daily intake	β_12_	0.38 (0.30 − 0.46)	< 0.0001^*^	1.47 (1.36 − 1.59)	1.50 (1.42 − 1.58)
*Pinelliae tuber* (PT) daily intake	β_13_	0.05 (0.01 − 0.09)	0.0204^*^	1.05 (1.01 − 1.10)	1.15 (1.11 − 1.18)
Sex (Male)	β_14_	0.37 (0.30 − 0.45)	< 0.0001^*^	1.45 (1.34 − 1.57)	1.55 (1.45 − 1.67)
Age (≥ 60 years)	β_15_	0.65 (0.55 − 0.75)	< 0.0001^*^	1.92 (1.74 − 2.11)	1.94 (1.77 − 2.13)
SR daily intake*Age (≥ 60 years)	β_16_	0.17 (0.10 − 0.25)	< 0.0001^*^	3.35 (3.12 − 3.60)	
SR daily intake*PT daily intake	β_17_	-0.04 (− 0.06 − -0.01)	0.0043^*^	1.49 (1.46 − 1.53)	

*CI* Confidence interbal

^*^ *p*-value < 0.05

The ROC curve of PS determined the accuracy of the model predictions for treatment allocation. The areas under the ROC curves for the SR, BR, and PT were 0.77708, 0.72868, and 0.79195, respectively (data not shown). In the presence or absence of the SR, the SMD decreased for all factors (Table 4). In contrast, the SMD for age increased with the presence or absence of BR, and the SMD for sex increased with the presence or absence of PT (Table 4). Before PS matching, the crude ROR (95% CI) for SR, BR, and PT were 2.79 (2.42–3.21), 1.55 (1.34–1.79), and 1.97 (1.69–2.29), respectively. After PS matching, the RORs for the SR, BR, and PT were 2.84 (2.25–3.58), 0.89 (0.73–1.09), and 1.06 (0.84–1.35), respectively.

**Table 4:** Comparison of the number of reports for each factor before and after propensity score matching

	Before propensity score matching				After propensity score matching			
	Contain	Not Contain	p	Standard mean differences	Contain	Not Contain	p	Standard mean differences
*Scutellariae radix* (SR)	(*n* = 1,554)	(*n* = 2,686)			(*n* = 832)	(*n* = 832)		
Sex			0.2887	0.0339			0.6401	0.0229
Male	567 (0.3649)	1024 (0.3812)			283 (0.3401)	274 (0.3293)		
Female	987 (0.6351)	1662 (0.6188)			549 (0.6599)	558 (0.6707)		
Age			< 0.0001^*^	0.3508			0.1045	0.0796
≥ 60	862 (0.5547)	1936 (0.7208)			466 (0.5601)	433 (0.5204)		
< 60	692 (0.4453)	750 (0.2792)			366 (0.4399)	399 (0.4796)		
SR (g)	-	-			-	-		
BR (g)	3.05 ± 3.13	0.38 ± 0.80	< 0.0001^*^	1.1688	0.39 ± 1.06	0.36 ± 0.89	0.4580	0.0307
PT (g)	2.29 ± 2.44	0.54 ± 1.59	< 0.0001^*^	0.8498	0.88 ± 1.81	0.78 ± 1.81	0.2758	0.0552
*Bupleuri radix* (BR)	(*n* = 1,390)	(*n* = 2850)			(*n* = 993)	(*n* = 993)		
Sex			0.2627	0.0367			0.6046	0.0232
Male	505 (0.3633)	1086 (0.3811)			341 (0.3434)	352 (0.3545)		
Female	885 (0.6367)	1764 (0.6189)			652 (0.6566)	641 (0.6455)		
Age (years)			0.4587	0.0243			< 0.0001^*^	0.2173
≥ 60	928 (0.6676)	1870 (0.6561)			660 (0.6647)	757 (0.7623)		
< 60	462 (0.3324)	980 (0.3439)			333 (0.3353)	236 (0.2377)		
SR (g)	1.61 ± 1.42	0.51 ± 0.98	< 0.0001^*^	0.9016	1.02 ± 1.25	1.00 ± 1.31	0.7578	0.0156
BR (g)	-	-			-	-		
PT (g)	2.16 ± 2.42	0.70 ± 1.78	< 0.0001^*^	0.6873	1.12 ± 1.83	0.94 ± 1.97	0.0382^*^	0.0947
*Pinelliae tuber* (PT)	(*n* = 1,086)	(*n* = 3,154)			(*n* = 640)	(*n* = 640)		
Sex			0.0457^*^	0.0700			0.0475^*^	0.1109
Male	435 (0.4006)	1156 (0.3665)			252 (0.3938)	287 (0.4484)		
Female	651 (0.5994)	1998 (0.6335)			388 (0.6063)	353 (0.5516)		
Age			< 0.0001^*^	0.1931			0.6874	0.0225
≥ 60	642 (0.5912)	2156 (0.6836)			398 (0.6219)	391 (0.6109)		
< 60	444 (0.4088)	998 (0.3164)			242 (0.3781)	249 (0.3891)		
SR (g)	1.88 ± 1.32	0.52 ± 1.02	< 0.0001^*^	1.1530	1.19 ± 1.24	1.30 ± 1.46	0.1573	0.0812
BR (g)	3.59 ± 3.22	0.58 ± 1.32	< 0.0001^*^	1.2232	1.59 ± 2.47	1.44 ± 2.29	0.2428	0.0630
PT (g)	-	-			-	-		

*p-value < 0.05

**Fig. 3:**
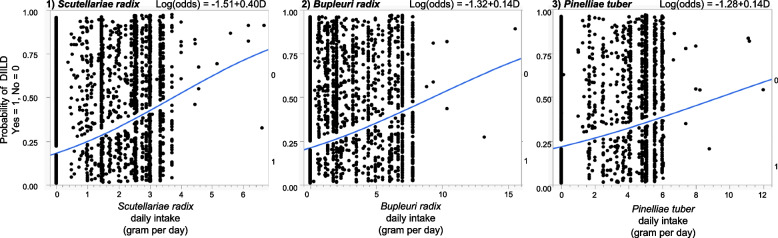
Logistic plots of daily intake (gram per day) and the onset of drug-induced interstitial lung disease (yes = 1, no = 0)

**Fig. 4:**
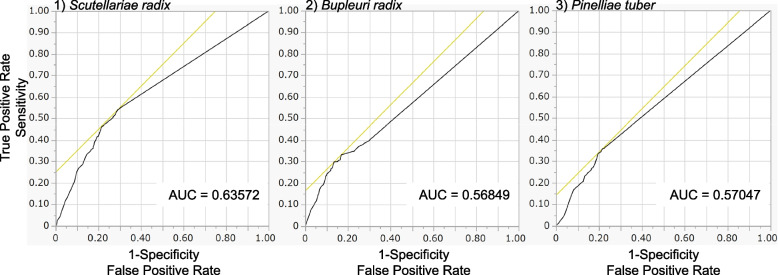
Receiver operating characteristic curve of SR, BR, and PT

We analyzed the relationship between the daily intake of crude drugs and the onset of DIILD (Table 5 and Fig. 3). The estimated β_21_ values (95% CI) for SR, BR, and PT were 0.40 (0.35–0.46) (*p* < 0.0001), 0.14 (0.11–0.17) (*p* < 0.0001), and 0.14 (0.10–0.17) (*p* < 0.0001), respectively (Table 5). The slope of the graph was the largest for SR (Table 5 (β_21_ = 0.40) and Fig. 3). According to the ROC curves for SR (AUC = 0.63572), BR (AUC = 0.56849), and PT (AUC = 0.57047), the cut-off values (g/day) were 0.96, 3.30, and 1.60, respectively (Fig. 4).

**Table 5:** The statistics of logistic regression of daily intake and onset of drug-induced interstitial lung disease

		Estimate (95% CI)		*p*-value (Prob > ChiSq)	Crude reporting odds ratio (95% CI)
*Scutellariae radix* (SR)	Intercept	β_20_	-1.51 (-1.61 – -1.42)	< 0.0001^*^	
	SR daily intake	β_21_	0.40 (0.35 – 0.46)	< 0.0001^*^	1.50 (1.42–1.58)
*Bupleuri radix* (BR)	Intercept	β_20_	-1.32 (-1.40 – -1.24)	< 0.0001^*^	
	BR daily intake	β_21_	0.14 (0.11 – 0.17)	< 0.0001^*^	1.15 (1.12–1.18)
*Pinelliae tuber* (PT)	Intercept	β_20_	-1.28 (-1.37 – -1.20)	< 0.0001^*^	
	PT daily intake	β_21_	0.14 (0.10 – 0.17)	< 0.0001^*^	1.15 (1.11–1.18)

*CI* Confidence interval

^*^ *p*-value < 0.05

**Fig. 5:**
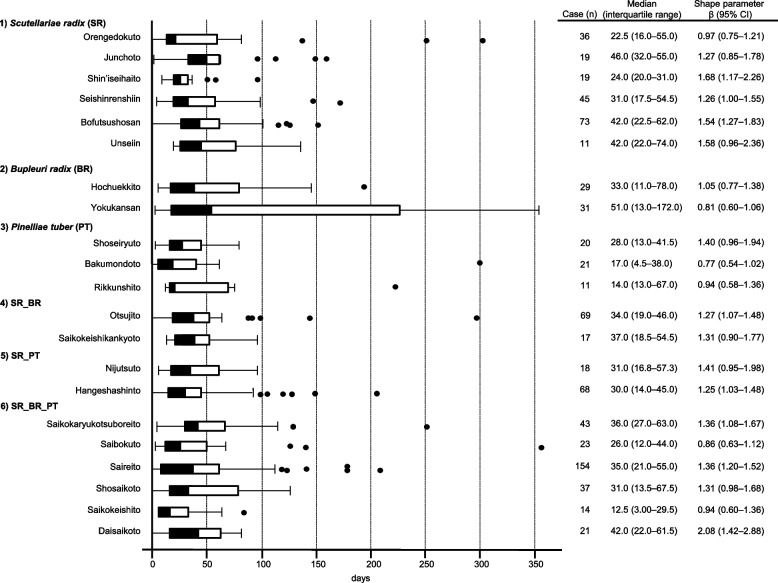
Time-to-onset profiles of drug-induced interstitial lung disease

**Fig. 6:**
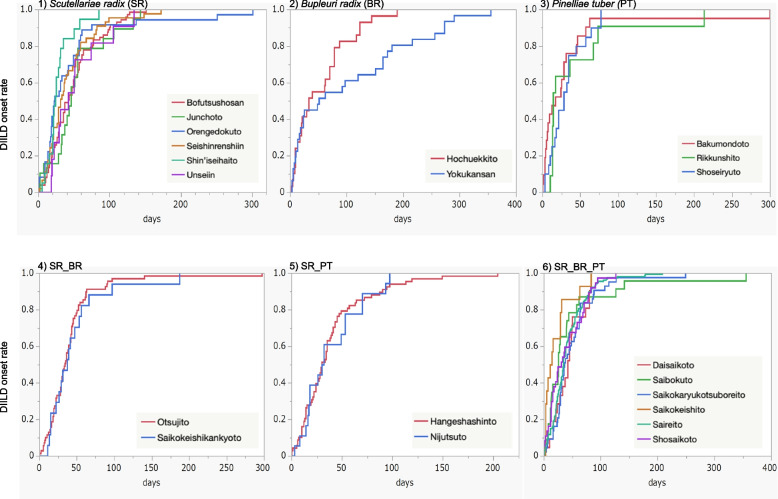
Kaplan–Meier curve plot for each group (SR, BR, PT, SR_BR, SR_PT, and SR_BR_PT)

To analyze the time-to-onset of DIILD, we evaluated 21 herbal medicines used in > 10 reported cases. The median duration (days) (interquartile range) to the onset of DIILD was 36.0 (27.0–63.0) days for Saikokaryukotsuboreito (containing SR_BR_PT), 35.0 (21.0–55.0) days for Saireito (containing SR_BR_PT), and 31.0 (13.5–67.5) days for Shosaikoto (containing SR_BR_PT) (Figs. 5 and 6). The Weibull shape parameters β (95% CI) of Saikokaryukotsuboreito (containing SR_BR_PT), Saireito (containing SR_BR_PT), and Shosaikoto (containing SR_BR_PT) were 1.36 (1.08–1.67), 1.36 (1.20–1.52), and 1.31 (0.98–1.68), respectively.

The underlying diseases of patients with DIILD are summarized in Table 6); the number of reported cases of hypertension and constipation in the underlying disease of SR was 160 and 81, respectively. The number of reported cases of hypertension and dementia in the underlying diseases of BR were 161 and 107, respectively. The number of reported cases of hypertension and gastroesophageal reflux disease among the underlying diseases of PT were 87 and 28, respectively. The number of reported cases of hemorrhoids and hypertension in the underlying diseases of SR_BR were 60 and 33, respectively. The number of reported cases of hypertension and periarthritis in the underlying diseases of SR_PT were 33 and 21, respectively. The number of reported cases of diabetes and depression in the underlying diseases of SR_BR_PT were 40 and 33, respectively.

**Table 6:** The underlying diseases of the patients with DIILD

Crude drug component	Underlying disease	n (%)
*Scutellariae radix* (SR)	Hypertension	160 (11.29)
	Constipation	81 (5.72)
	Obesity	65 (4.59)
	Hyperlipidemia	59 (4.16)
	Diabetes	35 (2.47)
	Asthma	29 (2.05)
	Alcohol intake	28 (1.98)
	Dyslipidemia	24 (1.69)
	Benign prostatic hypertrophy	22 (1.55)
	Atopic dermatitis	21 (1.48)
	Insomnia	21 (1.48)
	Others	872 (61.54)
*Bupleuri radix* (BR)	Hypertension	161 (8.81)
	Dementia	107 (5.86)
	Alzheimer’s disease	64 (3.50)
	Insomnia	51 (2.79)
	Constipation	48 (2.63)
	Depression	38 (2.08)
	Menopausal symptoms	37 (2.03)
	Atrial fibrillation	33 (1.81)
	Hyperlipidemia	33 (1.81)
	Diabetes	31 (1.70)
	Cerebral infarction	29 (1.59)
	Others	1195 (65.41)
*Pinelliae tuber* (PT)	Hypertension	87 (8.42)
	Gastro esophageal reflux disease	28 (2.71)
	Asthma	23 (2.23)
	Constipation	23 (2.23)
	Hyperlipidemia	22 (2.13)
	Insomnia	22 (2.13)
	Nasopharyngitis	22 (2.13)
	Allergic rhinitis	18 (1.74)
	Bronchitis	16 (1.55)
	Diabetes	16 (1.55)
	Atrial fibrillation	15 (1.45)
	Alcohol intake	14 (1.36)
	Gastritis	14 (1.36)
	Osteoporosis	12 (1.16)
	Upper respiratory tract infection	12 (1.16)
	Chronic gastritis	11 (1.07)
	Poor appetite	11 (1.07)
	Others	667 (64.57)
SR_BR	Hemorrhoid	60 (15.04)
	Hypertension	33 (8.27)
	Constipation	30 (7.52)
	Hyperlipidemia	15 (3.76)
	Gastro esophageal reflux disease	14 (3.51)
	Insomnia	7 (1.75)
	Acne	6 (1.50)
	Anxiety disorder	6 (1.50)
	Dyslipidemia	6 (1.50)
	Menopausal symptoms	6 (1.50)
	Asthma	5 (1.25)
	Hyperuricemia	5 (1.25)
	Osteoarthritis	5 (1.25)
	Others	201 (50.38)
SR_PT	Hypertension	33 (9.19)
	Periarthritis	21 (5.85)
	Gastro esophageal reflux disease	14 (3.90)
	Colonic cancer	9 (2.51)
	Stomatitis	8 (2.23)
	Diarrhea	7 (1.95)
	Hyperlipidemia	7 (1.95)
	Non-small cell lung cancer	7 (1.95)
	Dyslipidemia	6 (1.67)
	Benign prostatic hypertrophy	5 (1.39)
	Diabetes	5 (1.39)
	Insomnia	5 (1.39)
	Liver metastasis	5 (1.39)
	Lung metastasis	5 (1.39)
	Rectal cancer	5 (1.39)
	Others	217 (60.45)
SR_BR_PT	Hypertension	110 (8.46)
	Diabetes	40 (3.08)
	Depression	33 (2.54)
	Subdural hematoma	33 (2.54)
	Hyperlipidemia	28 (2.15)
	Insomnia	21 (1.62)
	Dizziness	20 (1.54)
	Sudden sensorineural hearing loss	19 (1.46)
	Asthma	18 (1.39)
	Anxiety disorder	16 (1.23)
	Alcohol intake	15 (1.15)
	Gastro esophageal reflux disease	15 (1.15)
	Non-smoker	15 (1.15)
	Others	917 (70.54)

## Discussion

AE signals for DIILD were detected in 59% of the herbal medicines containing SR, BR, and PT in the JADER database. We could not evaluate the effect of AEs on the incidence rates according to sex and age based on the crude ROR, as the crude ROR indicated an increased risk of AE reporting, but did not present the risk of AE occurrence in absolute terms. Multivariate logistic regression offers the advantage of controlling for covariates and can be used to analyze the use of interaction terms in more detail [31–33]. Our adjusted ROR results suggest an interaction between daily SR intake and age. Male sex and age ≥ 60 years were risk factors for DIILD [2]. Mortality rates in interstitial lung disease are highest in men and older adults [34]. In Japan, approximately 60% of patients using Japanese herbal medicines are over the age of 60 years [35–37]. This information is important for healthcare professionals.

Both the ROR (1.50 [1.42–1.58]; Table 5) and ROR (2.84 [2.25–3.58]) of SR after PS matching were higher than those of BR and PT. We observed that increased daily intake of SR, BR, and PT was associated with an increased incidence of DIILD and determined the dose response to crude drugs for DIILD onset. The inadvertent administration of herbal medicines is a risk factor for DIILD. The slope (β_21_) of SR was larger than that of the other drugs. This result may reflect the differences in the pharmacological actions of SR and other herbal medicines. However, further in vivo studies are required to confirm these results. Based on ROC curve analysis, if the daily intake of SR was greater than 0.96 g/day, this trend was more likely to appear than the following administration at lower doses.

Two mechanisms are involved in the pathogenesis of DIILD: direct dose-dependent toxicity and immune-mediated toxicity [38]. Both SR and BR are thought to cause lung injury [14]. SR mainly contains baicalein and baicalin as chemical ingredients, which may be associated with the onset of DIILD. A previous study suggested that baicalein induces apoptosis in human lung fibroblasts [39], which is essential for the growth and differentiation of type 2 alveolar epithelial cells [40], which are necessary for normal lung repair [41]. Pulmonary fibrosis is an interstitial lung disease caused by fibrosis due to remodeling associated with defective re-epithelization [42, 43]. Baicalein reduced the number of lung fibroblasts, negatively affecting the growth and differentiation of type 2 alveolar epithelial cells. Under these conditions, the lungs are not repaired properly, leading to fibrillation. Based on these findings, SR was considered a crude drug associated with DIILD. A high dose of baicalin (400–1600 mg/kg) induced fibrosis in Sprague–Dawley rats by activating the TGF-β/Smad signaling pathway [44]. According to the package inserts of Japanese herbal medicines, the daily dose of baicalein is estimated to be less than 200 mg/d [45]. The results from the animal studies cannot be extrapolated to patients; however, our cut-off value of 0.96 g/day may be worthy of further investigation.

Several studies of BR and PT have demonstrated dose-dependent toxicity in vitro and in organs such as the liver [46–51]. For PT, we observed a few reports on lymphocyte stimulation tests [8, 11–13]. Previous studies on BR and PT have reported pulmonary toxicity [11–13]. Our results did not demonstrate clear correlations between daily intake, BR, PT, and DIILD onset. The crude components of herbal medicines should be considered when determining their appropriateness for daily consumption.

More than 50% of DIILD cases associated with herbal medicine in all groups, except BR were observed within 50 days in the real-world data used in the time-to-onset analysis. Herbal medicines containing BR showed a longer time to DIILD onset. DIILD can occur after 180 days (six months) of administration of Orengedokuto (SR), Hochuekkito (BR), Yokukansan (BR), Bakumondoto (PT), Otsujito (SR_BR), Hangeshashinto (SR_PT), Saibokuto (SR_BR_PT), and Saireito (SR_BR_PT). This information may enable clinicians to perform early interventions to prevent DIILD progression. Herbal medicines (56% [9/16]) containing SR exhibited wear-off failure effects, and the rate of DIILD increased over time. SR may be cytotoxic because it is often expressed immediately if the expression mechanism is allergic, and delayed if it is cytotoxic [1, 38].

Our study has several limitations. SRS, such as JADER, are subject to overreporting, underreporting, missing data, exclusion of data from healthy individuals, lack of a denominator, and the presence of confounding factors. There was a lack of comparisons between groups. Moreover, it should be emphasized in SRS studies that disproportionality measures (crude ROR) do not allow for risk quantification. RORs offer a rough indication of the signal strength. In absolute terms, the ROR indicates an increased risk of AE reporting, and not the risk of AE occurrence. Comparing the intensity of risk based on measures, such as the crude ROR value, in a disproportionality analysis is not recommended [52].

The ROR is a pharmacovigilance index used by Japanese and Netherlands administrative authorities. In SRS studies, it is common practice to summarize the number of reports and RORs to provide an overview of the data (Table 1). Multiple logistic regression analysis and PS matching were used to avoid inappropriate data interpretation of the crude ROR for conventional signal detection. In this study, the adjusted ROR by multiple logistic regression analysis was described, and the ROR of the three herbal medicines were compared based on the adjusted ROR using the dataset after PS matching (Table 4). Several previous studies have compared RORs after matching backgrounds using PS matching, and we believe that our analysis method is valid. However, the effects of multivariate logistic regression and PS matching techniques are partial and subject to bias due to unmeasured confounders. Therefore, careful attention must be paid when interpreting results from the JADER database. Further epidemiological studies are required to determine the effects of these factors.

Depending on the analysis period of the time-to-onset profile, the hazard trends (increase, decrease, or constant) may differ [28]. Therefore, the follow-up duration must be carefully determined. To our knowledge, there is no gold standard for analyzing SRS research. The parameters were determined according to the dataset and the purpose of the study. In our previous research on DIILD of 110 drugs, including herbal medicines, we chose an analysis period of 730 days to focus on the onset of AEs within 2 years [4]. In the research, the median of “Saireito” and “Shosaikoto” were 35.0 and 33.0 days, respectively. Furthermore, the median time to onset of DIILD by anticancer drugs known to be cytotoxic is 262 days at most [53–55]. In this study, we set the analysis period to one year because the longer the observation period, the more unknown factors other than drugs may affect AEs. Furthermore, one limitation of the WSP tool is that several AE mechanisms are not time dependent.

According to the National Health and Nutrition Survey by the Ministry of Health, Labor, and Welfare, smoking is a risk factor for DIILD, and men exhibit a higher rate of smoking than women in Japan [56]. Smoking history was not evaluated in this study because of a lack of data in the JADER database. Patients with DIILD have various underlying diseases such as hypertension; therefore, the evaluation of DIILD risk due to these differences in patient backgrounds should be considered in the future.

Herbal medicines contain several crude drugs, and DIILD is caused by herbal medicines that do not contain SR, BR, or PT [57]. A difference in the amount of baicalin in herbal formulations among pharmaceutical companies, even for the same formula, has been reported, which was not considered in our study [45]. In this study, we selected three herbal medicines that have been widely recognized as risky, based on previous studies.

## Conclusions

We determined the possible risk and time-of-onset of DIILD caused by herbal medicines. We observed a correlation between crude drug intake and DIILD onset. Clinicians should exercise caution when prescribing herbal medicines and should carefully consider the timing and dose of administration. Additionally, inadvertent administration of herbal medicines should be avoided.

## Data Availability

Data used in this study are available from the following. links: https://www.pmda.go.jp/safety/info-services/drugs/adr-info/suspected-adr/0004.html.

## Abbreviations

DIILD: Drug-induced interstitial lung disease
SR: *Scutellariae* Radix
BR: *Bupleuri* radix
PT: *Pinelliae* tuber
SRS: Spontaneous reporting system
ROR: Reporting odds ratio
AE: Adverse event
JADER: Japanese Adverse Drug Event Report
PMDA: Pharmaceutical and Medical Devices Agency
PS: Propensity score
MedDRA: Medical Dictionary for Regulatory Activities
WSP: Weibull shape parameter
SMD: Standard mean difference
ROC: Receiver operating characteristic

## References

[1] Schwaiblmair M, Behr W, Haeckel T, Märkl B, Foerg W, Berghaus T (2012). Drug induced interstitial lung disease. Open Respir Med J.

[2] Ministry of Health, Labour and Welfare. The manual for handling disorders due to adverse drug reactions, interstitial lung disease. 2019. https://www.mhlw.go.jp/topics/2006/11/tp1122-1b.html. Accessed 15 Jun 2022.

[3] Podolanczuk AJ, Wong AW, Saito S, Lasky JA, Ryerson CJ, Eickelberg O (2021). Update in Interstitial lung disease 2020. Am J Respir Crit Care Med.

[4] Matsumoto K, Nakao S, Hasegawa S, Matsui T, Shimada K, Mukai R (2020). Analysis of drug-induced interstitial lung disease using the Japanese Adverse Drug Event Report database. SAGE Open Med.

[5] Tsutani K (2010). Kusuri ha risuku : Kampoyaku kara seiyoyaku wo miru. Jpn J Pharmacoepidemiol.

[6] Kuchta K, Cameron S (2022). Editorial: Kampo medicine in a modern context: ethnopharmacological perspectives. Front Pharmacol.

[7] Shimada Y, Fujimoto M, Nogami T, Watari H (2019). Adverse events associated with ethical kampo formulations: analysis of the Domestic Adverse-Event Data Reports of the Ministry of Health, Labor, and Welfare in Japan. Evid Based Complement Alternat Med.

[8] Tsukiyama K, Tasaka Y, Nakajima M, Hino J, Nakahama C, Okimoto N (1989). A case of pneumonitis due to Sho-saiko-to. Nihon Kyobu Shikkan Gakkai Zasshi.

[9] Itoh T, Fujimoto H, Umekawa K, Rensha K, Minami K, Shoji S (2006). A case of Sai-rei-to-induced pneumonitis. Nihon Kokyuki Gakkai Zasshi.

[10] Miyagawa T, Mochizuki Y, Nakahara Y, Kawamura T, Sasaki S, Tsukamoto H (2009). A case of drug-induced pneumonitis due to Sai-rei-to. Nihon Kokyuki Gakkai Zasshi.

[11] Katou K, Mori K (1999). Autoimmune hepatitis with drug-induced pneumonia due to Sho-saiko-to. Nihon Kokyuki Gakkai Zasshi.

[12] Okada Y, Watanabe K, Suzuki Y, Suzuki K, Ito G, Muranushi A (1999). A case of hepatitis and intersitial pneumonitis induce by Hange-shasin-to and Sho-saiko-to. Kampo Med.

[13] Yamamoto T, Tsutsui N, Kazawa T, Sasagawa M, Sato Y (2016). A case of drug-induced pneumonitis due to Ougon and Saiko involved in Sai-rei-to showing strong uptake of Ga scintigraphy. Niigata Igakkai Zasshi.

[14] Komiya K, Ishii H, Ohama M, Uchida M, Tsubone T, Iwashita T (2012). Sai-rei-to-induced lung injury: a case report and brief review of the literature. Intern Med.

[15] Enomoto Y, Nakamura Y, Enomoto N, Fujisawa T, Inui N, Suda T (2017). Japanese herbal medicine-induced pneumonitis: a review of 73 patients. Respir Investig.

[16] Ministry of Health, Labour and Welfare. Pharmaceutical and Food Safety Bureau. Pharmaceuticals and Medical Devices Safety Information No. 146. 1998. https://www.pmda.go.jp/safety/info-services/drugs/calling-attention/safety-info/0147.html. Accessed 18 May 2022.

[17] Ministry of Health, Labour and Welfare. Pharmaceutical and Food Safety Bureau. Pharmaceuticals and Medical Devices Safety Information No. 158. 2000. https://www.pmda.go.jp/safety/info-services/drugs/calling-attention/safety-info/0089.html. Accessed 18 May 2022.

[18] PMDA website. https://www.pmda.go.jp/safety/info-services/drugs/adr-info/suspected-adr/0004.html. 2004. Accessed 18 May 2022.

[19] Pharmaceuticals and Medical Devices Agency. Find Review reports, PI; 2022. https://www.pmda.go.jp/english/search_index.html. Accessed 15 Jun 2022.

[20] Japanese Maintenance Organization. Medical Dictionary for Regulatory Activities MedDRA, version 23.0; 2021. https://www.jmo.pmrj.jp. Accessed 15 Jun 2022.

[21] The international council for harmonisation of technical requirements for pharmaceuticals for human use (ICH), Introductory guide MedDRA Version 23.0. https://admin.meddra.org/sites/default/files/guidance/file/intguide_%2024_0_English.pdf Accessed 28 Feb. 2024.

[22] van Puijenbroek EP, Bate A, Leufkens HG, Lindquist M, Orre R, Egberts AC (2002). A comparison of measures of disproportionality for signal detection in spontaneous reporting systems for adverse drug reactions. Pharmacoepidemiol Drug Saf.

[23] Poluzzi E, Raschi E, Piccinni C, De Ponti F, Karahoca A. Data Mining Techniques in Pharmacovigilance: analysis of the publicly accessible FDA Adverse Event Reporting System (AERS). InTech. 2012. p.265–302. 10.5772/50095.

[24] Nakao S, Hasegawa S, Shimada K, Mukai R, Tanaka M, Matsumoto K (2020). Evaluation of anti-infective-related clostridium difficile-associated colitis using the Japanese Adverse Drug Event Report database. Int J Med Sci.

[25] Rosenbaum PR, Rubin DB (1983). The central role of the propensity score in observational studies for causal effects. Biometrika.

[26] Akimoto H, Oshima S, Negishi A, Ohara K, Ohshima S, Inoue N (2016). Assessment of the risk of suicide-related events induced by concomitant use of antidepressants in cases of smoking cessation treatment with varenicline and assessment of latent risk by the use of varenicline. PLoS One.

[27] Nakao S, Hasegawa S, Umetsu R, Shimada K, Mukai R, Tanaka M (2021). Pharmacovigilance study of anti-infective-related acute kidney injury using the Japanese Adverse Drug Event Report database. BMC Pharmacol Toxicol.

[28] Sauzet O, Carvajal A, Escudero A, Molokhia M, Cornelius VR (2013). Illustration of the Weibull shape parameter signal detection tool using electronic healthcare record data. Drug Saf.

[29] Sasaoka S, Matsui T, Hane Y, Abe J, Ueda N, Motooka Y (2016). Time-to-onset analysis of drug-induced long QT syndrome based on a spontaneous reporting system for adverse drug events. PLoS One.

[30] Nakao S, Hatahira H, Sasaoka S, Hasegawa S, Motooka Y, Ueda N (2017). Evaluation of Drug-Induced Photosensitivity Using the Japanese Adverse Drug Event Report (JADER) Database. Biol Pharm Bull.

[31] van Puijenbroek EP, Egberts AC, Meyboom RH, Leufkens HG (1999). Signalling possible drug–drug interactions in a spontaneous reporting system: delay of withdrawal bleeding during concomitant use of oral contraceptives and itraconazole. Br J Clin Pharmacol.

[32] van Puijenbroek EP, Egberts AC, Heerdink ER, Leufkens HG (2000). Detecting drug–drug interactions using a database for spontaneous adverse drug reactions: an example with diuretics and non-steroidal anti-inflammatory drugs. Eur J Clin Pharmacol.

[33] Qian Y, Ye X, Du W, Ren J, Sun Y, Wang H (2010). A computerized system for detecting signals due to drug–drug interactions in spontaneous reporting systems. Br J Clin Pharmacol.

[34] Montesi SB, Fisher JH, Martinez FJ, Selman M, Pardo A, Johannson KA (2020). Update in Interstitial Lung Disease 2019. Am J Respir Crit Care Med.

[35] Cho SJ, Stout-Delgado HW (2020). Aging and Lung Disease. Annu Rev Physiol.

[36] Selman M, Pardo A (2014). Revealing the pathogenic and aging-related mechanisms of the enigmatic idiopathic pulmonary fibrosis. An integral model. Am J Respir Crit Care Med.

[37] Katayama K, Yoshino T, Munakata K, Yamaguchi R, Imoto S, Miyano S (2013). Prescription of kampo drugs in the Japanese health care insurance program. Evid Based Complement Alternat Med.

[38] Matsuno O (2012). Drug-induced interstitial lung disease: mechanisms and best diagnostic approaches. Respir Res.

[39] Liu ZL, Tanaka S, Horigome H, Hirano T, Oka K (2002). Induction of apoptosis in human lung fibroblasts and peripheral lymphocytes in vitro by Sho-saiko-to derived phenolic metabolites. Biol Pharm Bull.

[40] Barkauskas CE, Cronce MJ, Rackley CR, Bowie EJ, Keene DR, Stripp BR (2013). Type 2 alveolar cells are stem cells in adult lung. J Clin Invest.

[41] Adamson IY, Hedgecock C, Bowden DH (1990). Epithelial cell-fibroblast interactions in lung injury and repair. Am J Pathol.

[42] Antoniou KM, Margaritopoulos GA, Tomassetti S, Bonella F, Costabel U, Poletti V (2014). Interstitial lung disease. Eur Respir Rev.

[43] Burgoyne RA, Fisher AJ, Borthwick LA (2021). The role of epithelial damage in the pulmonary immune response. Cells.

[44] Cai Y, Ma W, Xiao Y, Wu B, Li X, Liu F (2017). High doses of baicalin induces kidney injury and fibrosis through regulating TGF-β/Smad signaling pathway. Toxicol Appl Pharmacol.

[45] Shii T, Kuroda M, Shamoto N, Mimaki Y (2020). An analysis of the ingredients in decoctions and extracts of Kampo medicines: amounts of baicalin and baicalein in Kampo medicines containing Scutellariae radix. Nihon Ronen Igakkai Zasshi.

[46] Li X, Li X, Huang N, Liu R, Sun R (2018). A comprehensive review and perspectives on pharmacology and toxicology of saikosaponins. Phytomedicine.

[47] Wang Q, Zheng XL, Yang L, Shi F, Gao LB, Zhong YJ (2010). Reactive oxygen species-mediated apoptosis contributes to chemosensitization effect of saikosaponins on cisplatin-induced cytotoxicity in cancer cells. J Exp Clin Cancer Res.

[48] Huang W, Sun R, Zhang Z (2010). "Dose-time-toxicity" relationship study on hepatotoxicity caused by multiple dose of total Bupleurum saponin crude extracts to rats. Zhongguo Zhong Yao Za Zhi.

[49] Zhang X, Cai Y, Wang L, Liu H, Wang X (2015). Optimization of processing technology of Rhizoma Pinelliae Praeparatum and its anti-tumor effect. Afr Health Sci.

[50] Zhao X, Zhao J, Hu R, Yao Q, Zhang G, Shen H (2017). Ruanjian Sanjie decoction exhibits antitumor activity by inducing cell apoptosis in breast cancer. Oncol Lett.

[51] Zhang ZH, Zhao YY, Cheng XL, Dai Z, Zhou C, Bai X (2013). General toxicity of Pinellia ternata (Thunb.) Berit. in rat: a metabonomic method for profiling of serum metabolic changes. J Ethnopharmacol.

[52] Montastruc JL, Sommet A, Bagheri H, Lapeyre-Mestre M (2011). Benefits and strengths of the disproportionality analysis for identification of adverse drug reactions in a pharmacovigilance database. Br J Clin Pharmacol.

[53] Komada F, Nakayama Y, Takara K (2018). Analysis of time-to-onset and onset-pattern of interstitial lung disease after the administration of monoclonal antibody agents. Yakugaku Zasshi.

[54] Komada F (2018). Analysis of time-to-onset of interstitial lung disease after the administration of small molecule molecularly-targeted drugs. Yakugaku Zasshi.

[55] Yonemori K, Hirakawa A, Kawachi A, Kinoshita F, Okuma H, Nishikawa T (2016). Drug induced interstitial lung disease in oncology phase I trials. Cancer Sci.

[56] Ministry of Health, Labour and Welfare. National Health and Nutrition Surve; 2019. https://www.mhlw.go.jp/bunya/kenkou/kenkou_eiyou_chousa.html. Accessed 15 Jun 2022.

[57] Fujita T, Nagakawa H, Izawa T, Okada T, Tanabe N, Takiguchi Y (2008). Case of Shakuyaku-kanzo-to-induced CD4 dominant pneumonitis diagnosed on day eight of the challenge test. Nihon Kokyuki Gakkai Zasshi.

